# Tsallis Entropy in Consecutive *k*-out-of-*n* Good Systems: Bounds, Characterization, and Testing for Exponentiality

**DOI:** 10.3390/e27090982

**Published:** 2025-09-20

**Authors:** Anfal A. Alqefari, Ghadah Alomani, Mohamed Kayid

**Affiliations:** 1 Department of Statistics and Operations Research, College of Science, Qassim University, Buraydah 51482, Saudi Arabia; aa.alqefari@qu.edu.sa; 2 Department of Mathematical Sciences, College of Science, Princess Nourah bint Abdulrahman University, Riyadh 11671, Saudi Arabia; gaalomani@pnu.edu.sa; 3 Department of Statistics and Operations Research, College of Science, King Saud University, Riyadh 11451, Saudi Arabia

**Keywords:** consecutive *k*-out-of-*n* good systems, Tsallis entropy, Shannon entropy, Rényi entropy, stochastic orders

## Abstract

This study explores the application of Tsallis entropy in evaluating uncertainty within the framework of consecutive *k*-out-of-*n* good systems, which are widely utilized in various reliability and engineering contexts. We derive new analytical expressions and meaningful bounds for the Tsallis entropy under various lifetime distributions, offering fresh insight into the structural behavior of system-level uncertainty. The approach establishes theoretical connections with classical entropy measures, such as Shannon and Rényi entropies, and provides a foundation for comparing systems under different stochastic orders. A nonparametric estimator is proposed to estimate the Tsallis entropy in this setting, and its performance is evaluated through Monte Carlo simulations. In addition, we develop a new entropy-based test for exponentiality, building on the distinctive properties of system lifetimes. So, Tsallis entropy serves as a flexible tool in both reliability characterization and statistical inference.

## 1. Introduction

In recent decades, the study of consecutive *k*-out-of-*n* systems has gained prominence due to their relevance across numerous engineering and industrial settings. These models effectively capture configurations found in applications such as communication relay networks, segmented pipeline systems, and complex assemblies used in high-energy physics. The operational behavior of such systems depends on the arrangement of their components and the logic that governs their collective functioning. A key configuration, known as the linear consecutive *k*-out-of-*n* good system, consists of *n* sequential components, each assumed to function independently and identically. The system remains operational provided that at least *k* consecutive components are functioning simultaneously. Classical series and parallel structures appear as limiting cases of this setup: the *n*-out-of-*n* case corresponds to a series system, while the *1*-out-of-*n* configuration resembles a parallel structure. The foundational contributions of researchers such as Jung and Kim [1], Shen and Zuo [2], Kuo and Zuo [3], Chung et al. [4], Boland and Samaniego [5], and Eryılmaz [6,7] have laid the groundwork for this domain. Among the various system configurations, linear arrangements that satisfy the condition 2*k* ≥ *n* are of particular significance. These systems offer a meaningful compromise between analytical tractability and practical relevance in reliability engineering. In such models, the lifetime of the *i*-th component is typically represented by *T_1_*, *T_2_*, …, *Tₙ*, where each component follows a common continuous lifetime distribution characterized by its probability density function (pdf) *f*(*t*), cumulative distribution function (cdf) *F*(*t*), and survival function *S*(*t*) = *P*(*T* > *t*). The overall system lifetime is denoted by $T_{k|n}$, representing the operational lifetime of a linear consecutive k-out-of-n good system. Eryılmaz [8] established that when the condition 2*k* ≥ n holds, the reliability function of such a system takes the form:

(1) $$S_{k|n}\left(t\right)=\left(n-k+1\right)S^{k}\left(t\right)-\left(n-k\right)S^{k+1}\left(t\right),\;t>0.$$

In the context of information theory, a key aim is to measure the uncertainty associated with probability distributions. This study examines the use of Tsallis entropy as a tool for evaluating such uncertainty in consecutive *k*-out-of-*n* good systems, where the components are assumed to have continuous lifetime distributions. It is important to highlight that Tsallis [9] stimulated a resurgence of interest in the generalized entropy measures, often referred to as Tsallis entropy, building on earlier work by Havrda and Charvát [10] and the independent development of similar concepts in ecology by Patil and Tallie [11]. It provides a flexible alternative to classical measures and is widely regarded for its capacity to capture non-extensive behavior. The Tsallis entropy of order β is mathematically defined as:

(2) $$H_{\beta }\left(T\right)=\frac{1}{1-\beta }\left[\int_{0}^{\infty }f^{\beta }\left(t\right)\;dt-1\right]=\frac{1}{1-\beta }\left[\int_{0}^{1}f^{\beta -1}\left(F^{-1}\left(u\right)\right)\;du-1\right],$$

$for\;all\;\beta \in \Theta =\left(0,1\right)\cup \left(1,\infty \right),$ where $F^{-1}(u)=inf\{t;F(t)\geq u\}$, for u∈[0,1], represents the quantile function of F(t). Of particular note, the Shannon differential entropy, introduced by Shannon [12], emerges as a special case of Tsallis entropy in the limit as β approaches 1. This foundational concept in information theory provides a baseline for measuring uncertainty under additive conditions. Mathematically, it is defined as:

(3) $$H\left(T\right)=\underset{\beta \to 1}{lim\;H_{\beta }\left(T\right)}=-\int_{0}^{\infty }\;f\left(t\right)\;\log f\left(t\right)\;dt.$$

An alternative and insightful expression of Tsallis entropy can be obtained by reformulating Equation (2) in terms of the hazard rate function. This representation provides a useful perspective in reliability analysis, particularly when examining lifetime distributions. The resulting form is given by:

(4) $$H_{\beta }\left(T\right)=\frac{1}{1-\beta }\left(\frac{1}{\beta }E\left[\lambda^{\beta -1}\left(T_{\beta }\right)\right]-1\right),$$

where λ(t)=f(t)/S(t) represents the hazard rate function, E(⋅) means the expectation, and $T_{\beta }$ follows a pdf given by:

(5) $$f_{\beta }\left(t\right)=\beta f\left(t\right)S^{\beta -1}\left(t\right),\;t>0,\;for\;all\;\beta >0.$$

Note that Equation (5) is known as the proportional hazards model in the literature and defines a general class of distributions, which are sometimes known as exponentiated survival distributions. The term proportional hazards model has been used widely to describe the relative risk or Cox model and in survival analysis because the parameter β controls the tail behavior of the distributions; for more details, see, e.g., Kalbfleisch and Prentice [13] and Lawless [14]. It has long been understood that entropic measures for continuous random variables often yield negative values across many distribution types. This observation holds for various entropy functionals, including those proposed by Shannon and Tsallis. While discrete random variables typically yield non-negative values under these measures, continuous ones can be negative. Moreover, both Tsallis and Shannon entropies share their invariance under location shifts and their sensitivity to scale transformations. Unlike the additive nature of Shannon entropy, which is only additive for independent random variables, Tsallis entropy is non-additive, making it a more general and adaptable tool in complex systems. Specifically, for two independent continuous random variables (T_1_, T_2_), the entropy satisfies the relation: $H_{\beta }\left(T_{1},T_{2}\right)=H_{\beta }\left(T_{1}\right)+H_{\beta }\left(T_{2}\right)+\left(1-\beta \right)H_{\beta }\left(T_{1}\right)H_{\beta }\left(T_{2}\right)$, however for Shannon entropy that the rightmost term vanishes because 1−β→0 as β→0, resulting in the classic additivity property when T_1_ and T_2_ are independent i.e., $H\left(T_{1},T_{2}\right)=H\left(T_{1}\right)+H\left(T_{2}\right).$ This non-additivity property is particularly relevant in the context of independent random variables, which underscores the versatility and theoretical strength of the Tsallis functional. When X and Y are dependent, we have $H(T_{1},T_{2})=H\left(T_{1}\right)+H\left(T_{2}\right)-I(X,Y),$ where I(X,Y) is the mutual information (see, e.g., Cover and Thomas [15]). A similar result holds for dependent random variables in the context of Tsallis entropy, as demonstrated by Màrius Vila et al. [16]. In the field of telecommunications, entropic measures such as those by Shannon and Tsallis help define the fundamental performance boundaries of communication systems. These include limits on data compression efficiency and transmission reliability. Shannon entropy, for instance, quantifies the average uncertainty or information content of messages from a given source. According to Shannon’s Source Coding Theorem, entropy defines the minimum average number of bits per symbol required for lossless encoding. Moreover, the Shannon–Hartley Theorem links entropy to the maximum achievable rate for reliable communication over a noisy channel. In essence, Shannon entropy is a cornerstone in information theory, setting the theoretical limits for the two most essential tasks in digital communication: efficient compression and robust transmission. For clarity, Table 1 presents our notation, common alternatives found in the literature, symbols, conceptual definitions, and the location of mathematical definitions.

The study of information-theoretic measures within the context of reliability systems and order statistics has attracted growing interest in recent decades. Several foundational works have shaped this domain, including contributions by Wong and Chen [17], Park [18], Ebrahimi et al. [19], Zarezadeh and Asadi [20], Toomaj and Doostparast [21], Toomaj [22], and Mesfioui et al. [23], among others. Building on this foundation, Alomani and Kayid [24] extended the analysis of Tsallis entropy to coherent and mixed systems, assuming independent and identically distributed (i.i.d.) component lifetimes. Further developments include Baratpour and Khammar [25], who investigated the entropy’s behavior with respect to order statistics and record values, and Kumar [26], who analyzed its relevance in the study of *r*-records. Kayid and Alshehri [27] provided notable advancements by deriving a closed-form expression for the lifetime entropy of consecutive k-out-of-n good systems. Their work also established a characterization result, proposed practical bounds, and introduced a nonparametric estimation method. Complementing these efforts, Kayid and Shrahili [28] focused on the fractional generalized cumulative residual entropy in similar systems, presenting a computational framework, establishing several preservation properties, and offering two nonparametric estimators supported by simulation-based evidence.

Building on previous studies, this work seeks to provide a more comprehensive understanding of how Tsallis entropy can be applied to analyze consecutive *k*-out-of-*n* good systems. We expand upon earlier findings by offering new insights into the structural properties of these systems, proposing improved bounding strategies, and developing estimation techniques tailored to their unique reliability characteristics. Although this study centers on consecutive systems with i.i.d. components, it is worth recognizing that more general binary systems, involving non-i.i.d. structures, have received significant attention in the literature. Notable contributions in this direction include the works of Tsallis et al. [29] and Hanel et al. [30,31], which examine how dependencies among components influence entropic formulations and their theoretical implications.

The structure of this paper is outlined as follows. In Section 2, we introduce a novel expression for the Tsallis entropy of consecutive *k*-out-of-*n* good systems, denoted by $T_{k|n}$, where component lifetimes are drawn from a general continuous distribution function *F*. This formulation is developed using the uniform distribution as a starting point. Due to the challenges involved in deriving closed-form results for Tsallis entropy in complex reliability settings, we also establish several analytical bounds, supported by illustrative numerical examples. Section 3 focuses on characterization results, highlighting key theoretical properties of Tsallis entropy in the setting of consecutive systems. In Section 4, we present computational validation of our findings and propose a nonparametric estimator specifically designed for evaluating system-level Tsallis entropy. The estimator’s performance is assessed using both simulated and empirical data. Finally, Section 5 summarizes the main conclusions and discusses their broader implications.

## 2. Tsallis Entropy of Consecutive k-out-of-n Good System

This section is structured into three parts. We begin with a brief overview of essential properties of Tsallis entropy and its connections to other well-known measures, such as Rényi and Shannon differential entropies. In the second part, we derive a closed-form expression for the Tsallis entropy in the context of consecutive *k*-out-of-*n* good systems and analyze its behavior with respect to different stochastic orderings. The final part introduces a series of analytical bounds that further clarify the entropy characteristics of these systems.

### 2.1. Results on Tsallis Entropy

In this paper, we consider a random variable, denoted by T, which is assumed to be absolutely continuous and nonnegative. A random variable is a mathematical construct used to represent the outcome of a random process, assigning numerical values to each possible outcome. In this context, *T* specifically represents the lifetime of a component, system, or living organism, meaning it quantifies the duration until a specific event occurs, such as the failure of a mechanical component, the breakdown of a system, or the death of an organism. The term absolutely continuous implies that the random variable *T* has a probability density function, allowing for a continuous range of possible values (e.g., any positive real number) rather than being restricted to discrete values. The nonnegative property ensures that T≥0, which is appropriate for modeling lifetimes, as time cannot be negative. This setup provides a flexible framework for analyzing the probabilistic behavior of lifetimes in various applications. Here, we present the relationship between Rényi and Tsallis entropy. For a non-negative random variable *T* with density function f(t), Rényi entropy introduces a tunable parameter β, allowing different aspects of the distribution’s uncertainty to be emphasized. This parameterized form enables more flexibility in analyzing the behavior of uncertainty across various probability models. It is formally defined as:

(6) $$R_{\beta }\left(T\right)=\frac{1}{1-\beta }\log\int_{0}^{\infty }f^{\beta }\left(t\right)\;dt,for\;all\;\beta >0,\beta \neq 1.$$

Both Tsallis and Rényi entropies serve as measures of deviation from uniformity, as they quantify the concentration of the probability distribution *f*. These entropy measures can take values across the extended real line, i.e., within the interval [−∞, ∞]. For an absolutely continuous, non-negative random variable *T*, it is established that $H_{\beta }\left(T\right)$ ≥ *H*(*T*) for all 0 < *β* < 1, and $H_{\beta }\left(T\right)$ ≤ *H*(*T*) for all *β* > 1. Furthermore, the relationship between Tsallis and Rényi entropies follows a similar pattern: $H_{\beta }\left(T\right)\geq R_{\beta }\left(T\right)$ when 0 < *β* < 1, and $H_{\beta }\left(T\right)\leq R_{\beta }\left(T\right)$ when *β* > 1. In the theorem that follows, we explore the connection between the Shannon differential entropy under the proportional hazards rate model, as defined in Equation (5), and the corresponding Tsallis entropy.

**Theorem 1.** 
*Let T be an absolutely continuous, non-negative random variable. Then, $H_{\beta }\left(T\right)\geq 1-H\left(T_{\beta }\right),\;$ for all 0<β<1 and $H_{\beta }\left(T\right)\leq 1-H\left(T_{\beta }\right),\;$ for all β>1*.

**Proof.**  By the log-sum inequality (see Cover and Thomas [15]), we have

(7) $$\begin{array}{c} \int_{0}^{\infty }f_{\beta }\left(t\right)\;\;\log\frac{\mathrm{\beta f}\left(t\right)S^{\beta -1}\left(t\right)}{f^{\beta }\left(t\right)}dt\geq \left(\int_{0}^{\infty }f_{\beta }\left(t\right)\;\;dt\right)\log\left(\frac{\int_{0}^{\infty }f_{\beta }\left(t\right)\;\;dt}{\int_{0}^{\infty }f^{\beta }\left(t\right)\;\;dt}\right)\\ \;\;\;\;\geq 1-\int_{0}^{\infty }f^{\beta }\left(t\right)\;\;dt, \end{array}$$

which implies

$$\int_{0}^{\infty }f(t)\;\log\lambda_{\beta }(t)dt\geq \int_{0}^{\infty }f^{\beta }(t)\;dt-1,$$

where $\lambda_{\beta }(t)=\beta \lambda (t)$ denotes the hazard rate function of $T_{\beta }$. By noting that

(8) $$\int_{0}^{\infty }f_{\beta }\left(t\right)\;\log\lambda_{\beta }\left(t\right)dt=1-H\left(T_{\beta }\right),$$

we get the results for all 1−β>0(1−β<0), and hence the theorem. □

### 2.2. Expression and Stochastic Orders

To derive the Tsallis entropy for a consecutive *k*-out-of-*n* good system, we begin by applying the probability integral transformation $W_{k|n}=F\left(T_{k|n}\right)$, where *F* is the continuous cumulative distribution function of the component lifetimes. Under standard assumptions, this transformation maps the system lifetime into a variable that follows a uniform distribution on the interval [0, 1]. Leveraging this property, we obtain an explicit form for the Tsallis entropy of the system lifetime $T_{k|n}$, assuming that the component lifetimes are independently and identically distributed. Based on Equation (1), the probability density function of $T_{k|n}$ is expressed as:

(9) $$f_{k|n}\left(t\right)=f\left(t\right)[k\left(n-k+1\right)S^{k-1}\left(t\right)-\left(k+1\right)\left(n-k\right)S^{k}\left(t\right)],t>0.$$

Furthermore, when 2k≥n, the pdf of $W_{k|n}=F\left(T_{k|n}\right)$ can be represented as follows:

(10) $$\rho_{k|n}(w)=k(n-k+1)(1-w{)}^{k-1}-(k+1)(n-k)(1-w{)}^{k},for\;all\;0<w<1.$$

We next state a key result that follows directly from the preceding analysis. As the proof closely parallels the argument used in Theorem 1 of Mesfioui et al. [23], it is omitted here for brevity.

**Proposition 1**.
*Let $T_{k|n:G}$ denote the system lifetime of a consecutive k-out-of-n good system, where 2k≥n. Then, for all β∈Θ, the Tsallis entropy of $T_{k|n:G}$ is given by:*

(11) $${\;\;H}_{\beta }\left(T_{k|n}\right)=\frac{1}{1-\beta }\left(\int_{0}^{1}\rho_{k|n:G}^{\beta }(w)\;\;f^{\beta -1}\left(F^{-1}(w)\right)dw-1\right).$$

In the next theorem, an alternative formulation of $H_{\beta }\left(T_{k|n}\right)$ is derived using Theorem 1 in conjunction with Newton’s generalized binomial theorem.

**Theorem 2.** 
*Under the conditions of Proposition 1, we get*

$$H_{\beta }\left(T_{k|n}\right)=\frac{1}{1-\beta }\left(\sum_{i=0}^{\infty }\left(\frac{\beta }{i}\right)\frac{[(k+1)(n-k){]}^{i+\beta }}{[k(k-n-1){]}^{i}}\frac{E\left[f^{\beta -1}\left(F^{-1}\left(Z_{i,k,\beta }\right)\right)\right]}{i+\beta (k-1)+1}-1\right),$$

*where $Z_{i,k,\beta }∼Beta(1,i+\beta (k-1)+1)$ and $\left(\frac{\beta }{i}\right)=\frac{\beta (\beta -1)\ldots (\beta -i+1)}{i!}$ for all β∈Θ*.

**Proof.**  By defining A=k(n−k+1) and B=(k+1)(n−k), and referring to (10) and (11), we find that

$$\begin{array}{l} \int_{0}^{1}\rho_{k|n}^{\beta }(w)f^{\beta -1}\left(F^{-1}(w)\right)\;\;dw\\ \;\;\;\;\;\;=\int_{0}^{1}(1-w{)}^{\beta k-1}(A-B(1-w){)}^{\beta }f^{\beta -1}\left(F^{-1}(w)\right)\;dw\\ \;\;\;\;=A^{\beta }\int_{0}^{1}z^{\beta (k-1)}{\left(1-\frac{B}{A}z\right)}^{\beta }f^{\beta -1}\left(F^{-1}(1-z)\right)\;\;dz,(taking\;z=1-w)\\ \;=A^{\beta }\sum_{i=0}^{\infty }\left(\frac{\beta }{i}\right){\left(\frac{B}{A}\right)}^{i}(-1{)}^{i}\;\;\int_{0}^{1}{\left(1-u\right)}^{i+\beta (k-1)}f^{\beta -1}\left(F^{-1}(u)\right)\;\;du,(taking\;u=1-z)\\ =A^{\beta }\sum_{i=0}^{\infty }\left(\frac{\beta }{i}\right){\left(\frac{\left(k+1)(n-k\right)}{k(k-n-1)}\right)}^{i}\frac{E\left[f^{\beta -1}\left(F^{-1}\left(Z_{i,k,\beta }\right)\right)\right]}{i+\beta (k-1)+1}\;\;, \end{array}$$

where the third equality follows directly from Newton’s generalized binomial series $(1-x{)}^{\beta }=\sum_{i=0}^{\infty }\left(\frac{\beta }{i}\right)(-1{)}^{i}x^{i}\;$. This result, in conjunction with Equation (11), complete the proof. □

To demonstrate the usefulness of the representation given in Equation (11), we consider the following illustrative example.

**Example 1.** 
*Consider a linear consecutive 2-out-of-4 good system whose lifetime is given by:*

$$T_{2|4}=max\left(min\left(T_{1},T_{2}\right),min\left(T_{2},T_{3}\right),min\left(,T_{3},T_{4}\right)\right).$$

*Let us assume that the component lifetimes are i.i.d. and follow the log-logistic distribution (known as the Fisk distribution in economics). The pdf of this distribution with the shape parameter* γ* and the scale parameter one is represented as follows:*

(12) $$f\left(t\right)=\frac{\gamma t^{\gamma -1}}{{\left(1+t^{\gamma }\right)}^{2}},t,\gamma >0.$$

*After appropriate algebraic manipulation, the following identity is obtained:*

$$f\left(F^{-1}(w)\right)=\gamma w^{\frac{\gamma -1}{\gamma }}(1-w{)}^{\frac{\gamma +1}{\gamma }},\mathrm{for}\;0<w<1.$$

*As a result of specific algebraic manipulations, we obtain the following expression for the Tsallis entropy:*

(13) $$H_{\beta }\left(T_{2|4}\right)=\frac{1}{1-\beta }[\gamma^{\beta -1}\int_{0}^{1}\rho_{2|4}^{\beta }(w)w^{\frac{\left(\gamma -1\right)\left(\beta -1\right)}{\gamma }}(1-w{)}^{\frac{\left(\gamma +1\right)\left(\beta -1\right)}{\gamma }}dw-1],for\;all\;\beta \in \Theta .$$

*Due to the complexity of deriving a closed-form expression, numerical techniques are used to explore how the Tsallis entropy* $H_{\beta }\left(T_{2|4}\right)$*varies with the parameters β and γ. The analysis focuses on the consecutive 2-out-of-4 good system and is conducted for values* β>1 *and* γ>1*, since the integral diverges when* 0<β<1 *or* 0<γ<1.

**Figure 1:**
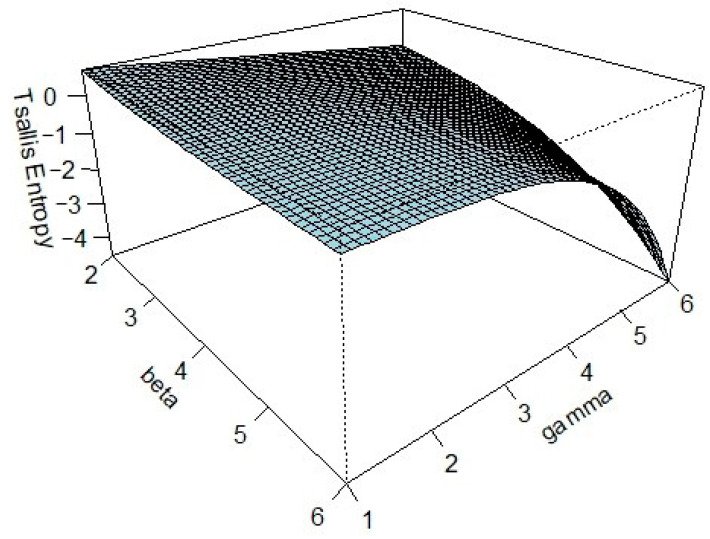
The plot of $H_{\beta }\left(T_{2|4}\right)$ with respect to β and γ as demonstrated in Example 1.

Figure 1 demonstrates that Tsallis entropy decreases as both β and γ increase. This behavior highlights the entropy’s sensitivity to changes in these parameters and emphasizes their influence on the system’s underlying uncertainty and information-theoretic profile.

**Definition 1.** 
*Assume two absolutely continuous nonnegative random variables $T_{1}$ and $T_{2}$ with pdfs $f_{1}$ and $f_{2}$, cdfs $F_{1}$ and $F_{2}$ and survival functions $S_{1}$ and $S_{2}$, respectively. Then, (i) T1≤T2 disp  i.e., $T_{1}$ is smaller than or equal to $T_{2}$ in the dispersive order, if and only if $f_{1}\left(F_{1}^{-1}(w)\right)\geq f_{2}\left(F_{2}^{-1}(w)\right)$ for all 0<w<1; (ii) T1≤T2 hr i.e., $T_{1}$ is smaller than $T_{2}$ in the hazard rate order if $S_{2}(t)/S_{1}(t)$ is increasing for all t>0; (iii) $T_{1}$ has a decreasing failure rate (DFR) property if $f_{1}(t)/S_{1}(t)$ is decreasing in t>0*.

For a thorough discussion of stochastic ordering concepts, readers are referred to the seminal work of Shaked and Shanthikumar [32]. The next theorem follows directly from the representation established in Equation (11).

**Theorem 3.** 
*Let $T_{k|n}^{1}$ and $T_{k|n}^{2}$ be the lifetimes of two consecutive k-out-of-n good systems having n i.i.d. component lifetimes with cdfs $F_{1}$ and $F_{2}$, respectively. If T1≤T2 disp , then*

$$H_{\beta }\left(T_{k|n}^{1}\right)\leq H_{\beta }\left(T_{k|n}^{2}\right),for\;all\;\beta \in \Theta .$$

**Proof.**  If T1≤T2 disp , then for all β≥(≤)1, we have

$$\begin{array}{c} \left(1-\beta \right)H_{\beta }\left(T_{k|n}^{1}\right)\;=\int_{0}^{1}\rho_{k|n}^{\beta }\left(w\right)f_{1}^{\beta -1}\left(F_{1}^{-1}\left(w\right)\right)dw-1\;\;\;\;\;\;\;\;\leq \\ \left(\geq \right)\int_{0}^{1}\rho_{k|n}^{\beta }\left(w\right)f_{2}^{\beta -1}\left(F_{2}^{-1}\left(w\right)\right)\;\;dw-1=H_{\beta }\left(T_{k|n}^{2}\right). \end{array}$$

This yields that $H_{\beta }\left(T_{k|n}^{1}\right)\leq H_{\beta }\left(T_{k|n}^{2}\right),$ for all β∈Θ, and this completes the proof. □

The following result formally establishes that, among consecutive *k*-out-of-*n* good systems whose components possess the decreasing failure rate (DFR) property, the series system attains the minimum Tsallis entropy.

**Proposition 2.** 
*Let $T_{k|n}$ denote the lifetime of a consecutive k-out-of-n good system, comprising n i.i.d. components that exhibit the DFR property. Then, for 2k≥n, and for all β∈Θ,*

*(i)*  *it holds that $H_{\beta }\left(T_{1:n}\right)\leq H_{\beta }\left(T_{k|n}\right)$*.
*(ii)*  *it holds that $H_{\beta }\left(T_{1:r}\right)\leq H_{\beta }\left(T_{k|n}\right)$*.

**Proof.**  (i) It is easy to see that T1:n≤Tk∣nhr. Furthermore, if T exhibits the DFR property, then it follows that $T_{1:n}$ also possesses the DFR property. Due to Bagai and Kochar [33], it can be concluded that T1:n≤Tk∣n disp  which immediately obtain $H_{\beta }\left(T_{1:n}\right)\leq H_{\beta }\left(T_{k|n}\right)$ by recalling Theorem 3. (ii) Based on the findings presented in Proposition 3.2 of Navarro and Eryılmaz [34], it can be inferred that T1:r≤Tk∣nhr. Consequently, employing analogous reasoning to that employed in Part (i) leads to the acquisition of similar results. □

An important application of Equation (11) is in comparing the Tsallis entropy of consecutive *k*-out-of-*n* good systems with independent components drawn from different lifetime distributions. This comparison is formally addressed in the following result.

**Proposition 3.** 
*Under the conditions of Theorem 3, if $H_{\beta }\left(T_{1}\right)\leq H_{\beta }\left(T_{2}\right)$ for all β>0, and ${inf}_{A_{1}}\;k_{k|n}(w)\geq {sup}_{A_{2}}\;k_{k|n}(w)$, for $A_{1}=\left\{w\in [0,1]:f_{1}\left(F_{1}^{-1}(w)\right)\leq f_{2}\left(F_{2}^{-1}(w)\right)\right\},\;A_{2}=\left\{v\in [0,1]:f_{1}\left(F_{1}^{-1}(w)\right)>f_{2}\left(F_{2}^{-1}(w)\right)\right\}$, then $H_{\beta }\left(T_{k|n}^{1}\right)\leq H_{\beta }\left(T_{k|n}^{2}\right)$ for all 2k≥n and β∈Θ.*

**Proof.**  Given that $H_{\beta }\left(T_{1}\right)\leq H_{\beta }\left(T_{2}\right)$ for all β>0, Equation (2) implies

(14) $$H_{\beta }\left(T_{2}\right)-H_{\beta }\left(T_{1}\right)=\frac{1}{1-\beta }\int_{0}^{1}\zeta_{\beta }\left(w\right)dw\geq 0,$$

where $\zeta_{\beta }\left(w\right)=f_{2}^{\beta -1}\left(F_{2}^{-1}\left(w\right)-f_{1}^{\beta -1}\left(F_{1}^{-1}\left(w\right)\right)\right),0<w<1$. Assuming β>1, based on Equation (11), we have

$$\begin{array}{c} \;\;H_{\beta }\left(T_{k|n}^{2}\right)-H_{\beta }\left(T_{k|n}^{1}\right)\\ \;\;=\frac{1}{1-\beta }\left[\int_{0}^{1}\;\;\rho_{k|n}^{\beta }\left(w\right)\zeta_{\beta }\left(w\right)dw\right]\\ =\frac{1}{1-\beta }\left[\int_{A_{1}}\rho_{k|n}^{\beta }(w)\zeta_{\beta }(w)\;dw+\int_{A_{2}}\rho_{k|n}^{\beta }(w)\zeta_{\beta }(w)\;\;dw\right]\left(since\;A_{1}\cup A_{2}=[0,1]\right)\\ \;\;\;\geq \frac{{\left({inf}_{v\in A_{1}}\;\rho_{k|n}(w)\right)}^{\beta }}{1-\beta }\int_{A_{1}}\zeta_{\beta }(w)\;dw+\frac{{\left({sup}_{v\in A_{2}}\;\rho_{k|n}(w)\right)}^{\beta }}{1-\beta }\int_{A_{2}}\zeta_{\beta }(w)\;\;dw\\ \;\;\geq \frac{{\left({sup}_{v\in A_{2}}\;\rho_{k|n}(w)\right)}^{\beta }}{1-\beta }\int_{0}^{1}\;\zeta_{\beta }(w)\;dw\geq 0. \end{array}$$

The first inequality holds because $\zeta_{\beta }(w)\geq 0$ in $A_{1}$ and $\zeta_{\beta }(w)<0$ in $A_{2}$ when β>1. The last inequality follows directly from Equation (14). Consequently, we have $H_{\beta }\left(T_{k|n}^{1}\right)\leq H_{\beta }\left(T_{k|n}^{2}\right)$ for 2k≥n, which completes the proof for the case β>1. The proof for the case 0<β≤1, follows a similar argument. □

The following example serves to illustrate the practical application of the preceding proposition.

**Example 2.** 
*Assume coherent systems with lifetimes $T_{2|3}^{1}=min\left(max\left(T_{1},T_{2}\right),max\left(T_{2},T_{3}\right)\right)$ and $T_{2|3}^{2}=min\left(max\left(Z_{1},Z_{2}\right),max\left(Z_{2},Z_{3}\right)\right)$, where $T_{1},T_{2},T_{3}$ are i.i.d. component lifetimes with a common cdf $F_{1}(t)=1-e^{-2t},t>0$, and $Z_{1},Z_{2},Z_{3}$ are i.i.d. component lifetimes with the common cdf $F_{2}(t)=1-e^{-6t},t>0$. We can easily confirm that $H_{\beta }\left(T\right)=-0.125$ and $H_{\beta }\left(Z\right)=-0.0416$, so $H_{\beta }\left(T\right)\leq H_{\beta }\left(Z\right)$. Additionally, since $A_{1}=[0,1)$ and $A_{2}=\{1\}$, we have ${inf}_{A_{1}}\;\rho_{2|3}(w)={sup}_{A_{2}}\;\rho_{2|3}(w)=0$. Thus Theorem 3 implies that $H_{\beta }\left(T_{2|3}^{1}\right)\leq H_{\beta }\left(T_{2|3}^{2}\right).$*

### 2.3. Some Bounds

In situations where closed-form expressions for Tsallis entropy are unavailable, particularly for systems with diverse lifetime distributions or a large number of components, bounding techniques offer a practical approach for approximating the entropy’s behavior over the system’s lifetime. This subsection explores the use of analytical bounds to characterize the Tsallis entropy of consecutive *k*-out-of-*n* good systems. In particular, we present the following theorem, which establishes a lower bound on the system’s Tsallis entropy. This bound provides valuable insights into the entropy structure under realistic conditions and supports a deeper understanding of system-level uncertainty.

**Lemma 1.** 
*Consider a nonnegative continuous random variable T with pdf f and cdf F such that M=f(m)<∞, where m=sup{x:f(t)≤M}, denotes the mode of the pdf f. Then, for 2k≥n, we have*

$$H_{\beta }\left(T_{k|n}\right)\geq M^{\beta -1}H_{\beta }\left(W_{k|n}\right)+\frac{1}{1-\beta }\left(M^{\beta -1}-1\right),\;for\;all\;\beta \in \Theta .$$

**Proof.**  By noting that $f\left(F^{-1}(w)\right)\leq M,0<w<1$, then for 0<β<1, we have $f^{\beta -1}\left(F^{-1}(w)\right)\geq$ $M^{\beta -1},0<w<1$. Now, the identity 1−β>0 implies that

$$\begin{array}{c} H_{\beta }\left(T_{k|n}\right)=\frac{1}{1-\beta }\left(\int_{0}^{1}\rho_{k|n}^{\beta }(w)h^{\beta -1}\left(H^{-1}(w)\right)dw-1\right)\geq \frac{1}{1-\beta }\left(M^{\beta -1}\int_{0}^{1}\rho_{k|n}^{\beta }(w)dw-1\right)\\ =\frac{M^{\beta -1}}{1-\beta }\left(\int_{0}^{1}\rho_{k|n}^{\beta }(w)dw-1\right)+\frac{M^{\beta -1}}{1-\beta }\left(1-\frac{1}{M^{\beta -1}}\right)\\ \;\;\;\;\;\;\;=M^{\beta -1}S_{\beta }\left(W_{k|n}\right)+\frac{1}{1-\beta }\left(M^{\beta -1}-1\right), \end{array}$$

and hence the result. When β>1, then we have $f^{\beta -1}\left(F^{-1}(w)\right)\leq$ $M^{\beta -1},0<w<1$. Now, since 1−β<0, by using the similar arguments, we have the results. □

The following theorem presents a lower bound for the Tsallis entropy of $T_{k|n}$. This bound is expressed in terms of the Tsallis entropy of a consecutive *k*-out-of-*n* good system assuming uniformly distributed component lifetimes, and it incorporates the mode M of the original lifetime distribution.

**Example 3.** 
*Assume a linear consecutive k-out-of-n good system with lifetime*

(15) $${\;\;\;\;T}_{k|n}=max\left(T_{\left[1:k\right]},T_{\left[2:k+1\right]},\ldots ,T_{\left[n-k+1:n\right]}\right),$$

*where* $T_{\left[j:m\right]}=min\left(T_{j},\ldots ,T_{m}\right)$ *for* 1≤j<m≤n *. Let further that the lifetimes of the components are i.i.d. having the common mixture of two Pareto distributions with parameters* $\beta_{1}$ *and* $\beta_{2}$ *with pdf as follows:*

$$f(t)=\theta \beta_{1}x^{-\beta_{1}-1}+(1-\theta )\beta_{2}t^{-\beta_{2}-1},x\geq 1,0\leq \theta \leq 1,\beta_{1}>\beta_{2}>0.$$

*Given that the mode of this distribution is* m=1*, we can determine the mode value* M *as* $M=f(1)=\theta \beta_{1}+(1-\theta )\beta_{2}$. *Consequently, from Lemma 2, we get*

$$H_{\beta }\left(T_{k|n}\right)\geq {\left(\theta \beta_{1}+\left(1-\theta \right)\beta_{2}\right)}^{\beta -1}H_{\beta }\left(W_{r|n:G}\right)+\frac{1}{1-\beta }\left({\left(\theta \beta_{1}+\left(1-\theta \right)\beta_{2}\right)}^{\beta -1}-1\right),\mathrm{for}\;\mathrm{all}\;\beta >0.$$

The next theorem establishes bounds for the Tsallis entropy of consecutive *k*-out-of-*n* good systems by relating it to the Tsallis entropy of the individual component lifetimes.

**Theorem 4.** 
*When β>1(0<β<1), we have*

(16) $${\;\;\;H}_{\beta }\left(T_{k|n}\right)\geq \left(\leq \right)\rho_{k|n}^{\beta }\left(w^{⋆}\right)H_{\beta }\left(T\right)+\frac{1}{1-\beta }\left(\rho_{k|n}^{\beta }\left(w^{⋆}\right)-1\right),$$

*where* $\rho_{k|n}\left(w^{⋆}\right)$*where* $w^{⋆}=\frac{2n-3k+1}{\left(k+1)(n-k\right)}$.

**Proof.**  The mode of $k_{k|n}(w)$ is clearly observed as $w^{⋆}=\frac{2n-3k+1}{\left(k+1)(n-k\right)}$. As a result, we can establish that $\rho_{k|n}(w)\leq \rho_{k|n}\left(w^{⋆}\right)$ for 0<w<1. Therefore, for β>1(0<β<1), we can conclude that:

$$\begin{array}{c} H_{\beta }\left(T_{k|n}\right)=\frac{1}{1-\beta }\left[\int_{0}^{1}\rho_{k|n}^{\beta }(w){\left(f\left(F^{-1}(w)\right)\right)}^{\beta -1}dw-1\right]\geq (\\ \;\;\;\leq )\frac{1}{1-\beta }\left(\rho_{k|n}^{\beta }\left(w^{⋆}\right)\int_{0}^{1}{\left(f\left(F^{-1}(w)\right)\right)}^{\beta -1}dw-1\right)\\ =\frac{\rho_{k|n}^{\beta }\left(w^{⋆}\right)}{1-\beta }\left(\int_{0}^{1}{\left(f\left(F^{-1}(w)\right)\right)}^{\beta -1}dw-1\right)+\frac{\rho_{k|n}^{\beta }\left(w^{⋆}\right)}{1-\beta }\left(1-\frac{1}{\rho_{k|n}^{\beta }\left(w^{⋆}\right)}\right)\\ =\rho_{k|n}^{\beta }\left(w^{⋆}\right)H_{\beta }\left(T\right)+\frac{1}{1-\beta }\left(\rho_{k|n}^{\beta }\left(w^{⋆}\right)-1\right), \end{array}$$

and hence the theorem. □

To demonstrate the lower bound established in Theorem 5, we now consider its application to a consecutive *k*-out-of-*n* good system.

**Example 4.** 
*Let us consider a linear consecutive 10-out-of-18 good system with lifetime $T_{10|18}=$ $max\left(T_{\left[1:10\right]},T_{\left[2:11\right]},\ldots ,T_{\left[10:18\right]}\right)$, where $T_{\left[j:m\right]}=min\left(T_{j},\ldots ,T_{m}\right)$ for 1≤j<m≤18. In order to conduct the analysis, we assume that the lifetimes of the individual components in the system are i.i.d. according to a common standard exponential distribution. By performing a simple verification, we find that the optimal value $w^{⋆}$ is equal to 0.08, resulting in a corresponding value of $\rho_{10|18}\left(w^{⋆}\right)$ as 4.268. Utilizing Theorem 4, we can write*

$$\;\;\;H_{\beta }\left(T_{10|18}\right)\geq \left(\leq \right)4.268H_{\beta }\left(T\right)+3.268/(1-\beta ),\;\mathrm{for}\;\mathrm{all}\;\beta >1(0<\beta <1).$$

The next result establishes bounds for consecutive *k*-out-of-*n* good systems based on the hazard rate function of the component lifetimes.

**Proposition 4.** 
*Let $T_{i},i=1,2,\ldots ,n$, be the lifetimes of components of a consecutive k-out-of-n good systems with $T_{k|n}$ having the common failure rate function λ(t). If 2k≥n and β∈Θ, then*

$$\;\frac{1}{1-\beta }\left(\frac{(2k-n{)}^{\beta -1}}{\beta }E\left[\lambda^{\beta -1}\left(T_{k|n,\beta }^{12}\right)\right]-1\right)\leq H_{\beta }\left(T_{k|n}\right)\leq \frac{1}{1-\beta }\left(\frac{k^{\beta -1}}{\beta }E\left[\lambda^{\beta -1}\left(T_{k|n,\beta }^{12}\right)\right]-1\right)$$

*where* $T_{r|n:G,\beta }^{12}$ *has the pdf* $f_{k|n,\beta }^{12}(t)=\beta f_{k|n}(t)S_{k|n}^{\beta -1}(t)$ *, for* t>0.

**Proof.**  The hazard rate function of $T_{k|n}$ can be easily represented by $\lambda_{k|n}(t)=\psi_{k,n}(S(t))\lambda (t)$, where

$$\psi_{k,n}(z)=\frac{k\left(n-k+1\right)-\left(k+1\right)\left(n-k\right)z}{\left(n-k+1\right)-\left(n-k\right)z},0<z<1.$$

Since $\psi_{k,n}^{\prime }(z)<0$ for 2k≥n and 0<z<1, it follows that $\psi_{k,n}(z)$ is a monotonically decreasing function of z. Since $\psi_{k,n}(0)=k$ and $\psi_{k,n}(1)=2k-n$, we have $2k-n\leq \psi_{k,n}(S(t))\leq k$ for 0<S(t)<1, which implies that $2k-n\lambda (t)\leq \lambda_{k|n:G}(S(t))\leq k\lambda (t)$, for t>0. Combining this result with the relationship between Tsallis entropy and the hazard rate (as defined in Equation (4)) for β>1(0<β≤1), completes the proof. □

We now present an illustrative example to demonstrate the application of the preceding proposition.

**Example 5.** 
*Consider a linear consecutive 2-out-of-3 good system with lifetime $T_{2|3}=max\left(min\left(T_{1},T_{2}\right),min\left(T_{2},T_{3}\right)\right)$, where the component lifetimes $T_{i}$ are i.i.d. with an exponential distribution with the cdf $F(t)=1-e^{-\lambda t}$ for t>0. The exponential distribution has a constant hazard rate, $\lambda_{1}(t)=\lambda$, so, it follows that $E\left(\lambda \left(T_{2|3}^{12}\right)\right)=\lambda$. Applying Proposition 4 yields the bounds on the Tsallis entropy of the system as $-0.5\lambda \leq H_{\beta }\left(T_{2|3}\right)\leq -0.25\lambda$. Based on (11), one can compute the exact value as $H_{\beta }\left(T_{2|3}\right)=-0.35\lambda$ which falls within the bounds.*

The next theorem holds under the condition that the expected value of the squared hazard rate function of *T* exists.

**Theorem 5.** 
*Under the conditions of Proposition 4 such that $E\left(\lambda^{2}(T)\right)<\infty$, for 2k≥n and β>1(0<β≤1), it holds that*

$$\;H_{\beta }\left(T_{k|n}\right)\geq \left(\leq \right)\frac{1}{1-\beta }\left(\sqrt{\Omega_{k,n,\beta }E\left(\lambda^{2\left(\beta -1\right)}\left(T\right)\right)}-1\right),\;\Omega_{k,n,\beta }=\int_{0}^{1}w^{2}\rho_{k|n}^{4}(w)\;dw.$$

**Proof.**  It is not hard to see that $f_{k|n}(t)=f(t)\rho_{k|n}(F(t))$, while its failure rate function is given by

$$\lambda_{k|n}\left(t\right)=\lambda \left(t\right)\frac{F\left(t\right)\rho_{k|n}\left(F\left(t\right)\right)}{S_{k|n}\left(t\right)},fort>0.$$

Thus, by (4) and the Cauchy-Schwarz inequality, we have

$$\begin{array}{l} \int_{0}^{\infty }\lambda_{k|n}^{\beta -1}(t)f_{k|n}(t)S_{k|n}^{\beta -1}(t)\;dt\\ \;\;\;\;\;\;=\int_{0}^{\infty }\lambda^{\beta -1}(t)\sqrt{f(t)}\sqrt{f(t)}F(t)f_{k|n}^{2}(F(t))\;\;dt\\ \;\;\;\;\;\;\leq {\left(\int_{0}^{\infty }\lambda^{2(\beta -1)}(t)f(t)\;\;dt\right)}^{1/2}{\left(\int_{0}^{\infty }\;{\left(F(t)\rho_{k|n}^{2}(F(t))\right)}^{2}h(t)\;dt\right)}^{1/2}\\ \;\;\;\;\;\;={\left(E\left(\lambda^{2(\beta -1)}(T)\right)\right)}^{1/2}{\left(\int_{0}^{1}w^{2}\rho_{k|n}^{4}(w)\;\;dw\right)}^{1/2}. \end{array}$$

In the last equality, we use the substitution w=F(t), and this completes the proof. □

## 3. Characterization Results

This section presents characterization results for consecutive *k*-out-of-*n* good systems based on Tsallis entropy. The analysis focuses on linear consecutive (n-i)-out-of-n good systems, n≥2i and i=0,1,…,n/2. We begin by recalling a lemma that relies on the Müntz–Szász theorem, as presented by Kamps [35].

**Lemma 3.** 
*For an integrable function ψ(t) on the finite interval (a,b) if $\int_{a}^{b}x^{n_{j}}\psi (x)\;dx=0,j\geq$ 1, then ψ(x)=0 for almost all x∈(a,b), where $\left\{n_{j},j\geq 1\right\}$ is a strictly increasing sequence of positive integers satisfying $\sum_{j=1}^{\infty }\frac{1}{n_{j}}\;=\infty$*.

It is worth pointing out that Lemma 3 is a well-established concept in functional analysis, stating that the sets $\left\{x^{n_{1}},x^{n_{2}},\ldots ;1\leq n_{1}<n_{2}<\cdots \right\}$ constitutes a complete sequence. Notably, Hwang and Lin [36] expanded the scope of the Müntz-Szász theorem for the functions $\left\{\phi^{n_{j}}(x),n_{j}\geq 1\right\}$, where ϕ(x) is both absolutely continuous and monotonic over the interval (a,b).

**Theorem 6.** 
*Let us assume two consecutives (n−i)-out-of-n good systems with lifetimes $T_{n-i|n}^{1}$ and $T_{n-i|n}^{2}$ consisting of n i.i.d. components with cdfs $F_{1}$ and $F_{2}$, and pdfs $f_{1}$ and $f_{2}$, respectively. Then $F_{1}$ and $F_{2}$ belong to the same family of distributions, but for a change in location, if and only if for a fixed i≥0,*

(17) $${\;\;\;\;\;H}_{\beta }\left(T_{n-i|n}^{1}\right)=H_{\beta }\left(T_{n-i|n}^{2}\right),for\;all\;n\geq 2i,\;and\;\beta \in \Theta .\;$$

**Proof.**  Since $F_{1}$ and $F_{2}$ are from the same location family, $F_{2}(y)=F_{1}(y-a)$, for all y≥a and a∈R. Thus

$$\begin{array}{c} H_{\beta }\left(T_{n-i|n}^{2}\right)\;=\frac{1}{1-\beta }\int_{a}^{\infty }f_{2,n-i|n}^{\beta }(y)\;\;dy=\frac{1}{1-\beta }\int_{a}^{\infty }f_{1,n-i|n}^{\beta }(y-a)\;\;dy\;\;\\ \;\;\;\;\;\;\;=\frac{1}{1-\beta }\int_{0}^{\infty }f_{1,n-i|n}^{\beta }\left(x\right)\;\;dx=H_{\beta }\left(T_{n-i|n}^{1}\right).\left(taking\;x=y-a\right), \end{array}$$

implies the necessity part. For the sufficiency part, for a consecutive (n−i)-out-of-n good system, Equation (10) gives

(18) $$\rho_{n-i|n:F}(w)=(n-i)(i+1)(1-w{)}^{n-i-1}-i(n-i+1)(1-w{)}^{n-i}$$

where 0<w<1,n≥2i and 0≤i≤n/2. By assumption $H_{\beta }\left(T_{n-i|n}^{1}\right)=H_{\beta }\left(T_{n-i|n}^{2}\right)$, we can write

(19) $$\begin{array}{c} \frac{1}{1-\beta }\left(\int_{0}^{1}\;\;\rho_{n-i|n}^{\beta }(w)f_{1}^{\beta -1}\left(F_{1}^{-1}(w)\right)dw-1\right)\\ \;\;\;\;\;\;\;\;=\frac{1}{1-\beta }\left(\int_{0}^{1}\;\;\rho_{n-i|n}^{\beta }(w)f_{2}^{\beta -1}\left(F_{2}^{-1}(w)\right)dw-1\right), \end{array}$$

or equivalently

(20) $$\int_{0}^{1}(1-w{)}^{\left(n-2i\right)\beta }\phi_{i,n,\beta }(w)\left(f_{1}^{\beta -1}\left(F_{1}^{-1}\left(w\right)\right)-f_{2}^{\beta -1}\left(F_{2}^{-1}\left(w\right)\right)\right)\;\;dw=0,$$

where

(21) $$\phi_{i,n,\beta }\left(w\right)={\left[(n-i)(i+1)(1-w{)}^{i+1}-i(n-i+1)w^{i}\right]}^{\beta },\;for\;0<w<1.$$

Applying Lemma 3 to the complete sequence $\left\{w^{(n-2i)\beta },n\geq 2i\right\}$ with

$$\psi \left(w\right)=\phi_{i,n,\beta }\left(w\right)\left(f_{1}^{\beta -1}\left(F_{1}^{-1}\left(w\right)\right)-f_{2}^{\beta -1}\left(F_{2}^{-1}\left(w\right)\right)\right),$$

yields the result that $f_{1}^{\beta -1}\left(F_{1}^{-1}(w)\right)=f_{2}^{\beta -1}\left(F_{2}^{-1}(w)\right),a.e.w\in \left(0,1\right),$ or $f_{1}\left(F_{1}^{-1}(w)\right)=f_{2}\left(F_{2}^{-1}(w)\right),\;0<w<1$. Consequently, $F_{1}$ and $F_{2}$ are part of the same distribution family, differing only in a location shift. □

Noting that a consecutive *n*-out-of-*n* good system corresponds to a classical series system, the following corollary provides a characterization of its Tsallis entropy.

**Corollary 1.** 
*Let $T_{n|n}^{1}$ and $T_{n|n}^{2}$ be two series systems having the common pdfs $f_{1}(t)$ and $f_{2}(t)$ and cdfs $F_{1}(t)$ and $F_{2}(t)$, respectively. Then $F_{1}$ and $F_{2}$ belong to the same family of distributions, but for a change in location, if and only if*

$$H_{\beta }\left(T_{n|n}^{1}\right)=H_{\beta }\left(T_{n|n}^{2}\right),\;for\;all\;n\geq 1\;and\;\beta \in \Theta .$$

An additional useful characterization is presented in the following theorem.

**Theorem 7.** 
*Under the conditions of Theorem 6, $F_{1}$ and $F_{2}$ belong to the same family of distributions, but for a change in location and scale, if and only if for a fixed i,*

(22) $$\frac{H_{\beta }\left(T_{n-i|n}^{1}\right)}{H_{\beta }(T)}=\frac{H_{\beta }\left(T_{n-i|n}^{2}\right)}{H_{\beta }\left(T_{2}\right)},\;for\;all\;n\geq 2i,\;and\;\beta \in \Theta .$$

**Proof.**  The necessity is trivial. To establish sufficiency, we leverage Equations (6) and (18) to derive

(23) $$\left(\frac{H_{\beta }\left(T_{n-i|n}^{1}\right)}{H_{\beta }\left(T_{1}\right)}\right)=\frac{1}{1-\beta }\left(\int_{0}^{1}\rho_{n-i|n:G}^{\beta }(w)\frac{f_{1}^{\beta -1}\left(F_{1}^{-1}(w)\right)}{H_{\beta }\left(T_{1}\right)}\;\;dw-1\right).$$

An analogous argument can be made for $H_{\beta }\left(T_{n-i|n}^{2}\right)/H_{\beta }\left(T_{2}\right)$. If relation (22) holds for two cdfs $F_{1}$ and $F_{2}$, then we can infer from Equation (23) that

(24) $$\int_{0}^{1}k_{n-i|n}^{\beta }\left(w\right)\frac{f_{1}^{\beta -1}\left(F_{1}^{-1}\left(w\right)\right)}{H_{\beta }\left(T_{1}\right)}dw=\int_{0}^{1}k_{n-i|n}^{\beta }\left(w\right)\frac{f_{2}^{\beta -1}\left(F_{2}^{-1}\left(w\right)\right)}{H_{\beta }\left(T_{2}\right)}dw.$$

Let us set $c=H_{\beta }\left(T_{2}\right)/H_{\beta }\left(T_{1}\right)$. By similar arguments as in Theorem 6, we have

$$\int_{0}^{1}w^{\left(n-2i\right)\beta }\phi_{i,n,\beta }(w)\left(cf_{1}^{\beta -1}\left(F_{1}^{-1}\left(w\right)\right)-f_{2}^{\beta -1}\left(F_{2}^{-1}\left(w\right)\right)\right)dw=0.$$

The proof follows similarly to Theorem 6. □

Applying Theorem 7 yields the following corollary.

**Corollary 2.** 
*Suppose the assumptions of Corollary 1,* $F_{1}$ *and* $F_{2}$ *belong to the same family of distributions, but for a change in location and scale, if and only if for a fixed* n,

$$\;\;\;\;\frac{H_{\beta }\left(T_{n|n}^{1}\right)}{H_{\beta }(T)}=\frac{H_{\beta }\left(T_{n|n}^{2}\right)}{H_{\beta }\left(T_{2}\right)},\;\mathrm{for}\;\mathrm{all}\;n\geq 1,\;\mathrm{and}\;\beta \in \Theta .$$

The following theorem characterizes the exponential distribution through Tsallis entropy within the framework of consecutive *k*-out-of-*n* good systems. This result serves as the theoretical basis for a newly proposed goodness-of-fit test for exponentiality, intended to be applicable across a wide variety of datasets. To establish this characterization, we begin by introducing the lower incomplete beta function, defined as:

$$B\left(t;a,b\right)=\int_{0}^{t}x^{a-1}(1-x{)}^{b-1}dx,0<t<1,$$

where a and b are positive real numbers. When t=0, this expression reduces to the complete beta function. We now present the main result of this section.

**Theorem 8.** 
*Let us assume that $T_{n-i|n}$ is the lifetime of the consecutive (n−i)-out-of-n good system having n i.i.d. component lifetimes with the pdf f and cdfF. Then T has an exponential distribution with the parameter λ if and only if for a fixed i≥0,*

(25) $$H_{\beta }\left(T_{n-i|n}\right)=\frac{[(n-i)(i+1){]}^{\beta (n-i+1)}}{[i(n-i+1){]}^{\beta (n-i)}}B\left(\frac{i(n-i+1)}{\left(n-i)(i+1\right)};\beta (n-i),\beta +1\right)\left[\beta H_{\beta }(T)+\frac{\beta }{1-\beta }\right]-\frac{1}{1-\beta },\;\;$$

*for all n≥2i, and* β∈Θ.

**Proof.**  Given an exponentially distributed random variable T, its Tsallis entropy, directly calculated using (6), is $H_{\beta }(T)=\frac{\lambda^{\beta -1}-\beta }{\beta (1-\beta )}$. Furthermore, since $f\left(F^{-1}(w)\right)=\lambda (1-w)$, application of Equation (11) yields:

$$\begin{array}{c} H_{\beta }\left(T_{n-i|n}\right)=\frac{1}{1-\beta }\left[\int_{0}^{1}\rho_{n-i|n}^{\beta }\left(w\right)f^{\beta -1}\left(F^{-1}\left(w\right)\right)dw-1\right]\\ \;\;=\frac{\lambda^{\beta -1}}{1-\beta }\int_{0}^{1}\rho_{n-i|n}^{\beta }(w)(1-w{)}^{\beta -1}dw-\frac{1}{1-\beta }\\ \;\;\;\;\;\;\;\;\;=\left[\beta H_{\beta }(T)+\frac{\beta }{1-\beta }\right]\int_{0}^{1}\rho_{n-i|n}^{\beta }(w)(1-w{)}^{\beta -1}dw-\frac{1}{1-\beta }, \end{array}$$

for β>0. To derive the second term, let us set A=(n−i)(i+1) and B=i(n−i+1), upon recalling (10), it holds that

(26) $$\begin{array}{c} \int_{0}^{1}\rho_{n-i|n}^{\beta }(w)(1-w{)}^{\beta -1}dw=\int_{0}^{1}(1-w{)}^{\beta (n-i)-1}(A-B(1-w){)}^{\beta }\;\;dw\;\;\;\;\;\;\;\;\;\;\\ \;\;\;\;\;=A^{\beta }\int_{0}^{1}z^{\beta (n-i)-1}{\left(1-\frac{B}{A}z\right)}^{\beta }\;\;dz,(taking\;z=1-w)\\ \;\;\;\;\;\;\;=\frac{A^{\beta (n-i+1)}}{B^{\beta (n-i)}}\int_{0}^{\frac{B}{A}}u^{\beta (n-i)-1}(1-u{)}^{\beta }\;\;du,\left(taking\;u=\frac{B}{A}z\right)\\ \;\;\;\;=\frac{A^{\beta (n-i+1)}}{B^{\beta (n-i)}}B\left(\frac{B}{A};\beta (n-i),\beta +1\right),\;\;\;\;\;\;\;\;\;\;\;\;\;\;\;\;\;\;\;\;\;\;\;\;\;\;\;\;\;\;\;\;\;\;\;\;\;\;\;\;\;\;\;\; \end{array}$$

where the necessity is derived. To establish sufficiency, we assume that Equation (25) holds for a fixed value of r and assume that $D=\left[\beta H_{\beta }(T)+\frac{\beta }{1-\beta }\right]$. Following the proof of Theorem 6 and utilizing the result in Equation (26), we obtain the following relation

$$\;\;\;\frac{1}{1-\beta }\int_{0}^{1}\rho_{n-i|n}^{\beta }(w)f^{\beta -1}\left(F^{-1}(w)\right)\;dw=D\int_{0}^{1}\rho_{n-i|n}^{\beta }(w)(1-w{)}^{\beta -1}\;dw$$

which is equivalent to

(27) $$\int_{0}^{1}\rho_{n-i|n}^{\beta }(w)\left[f^{\beta -1}\left(F^{-1}\left(w\right)\right)-(1-\beta )D(1-w{)}^{\beta -1}\right]\;\;dw=0,$$

where $k_{n-i|n:G}(w)$ is defined in (18). Thus, it holds that

(28) $$\int_{0}^{1}(1-w{)}^{\left(n-2i\right)\beta }\phi_{i,n,\beta }(w)\left[f^{\beta -1}\left(F^{-1}\left(w\right)\right)-(1-\beta )D(1-w{)}^{\beta -1}\right]\;\;dw=0,$$

where $\phi_{i,n,\beta }(w)$ is defined in (21). Applying Lemma 3 to the function

$$\psi \left(w\right)=\phi_{i,n,\beta }\left(w\right)\left[f^{\beta -1}\left(F^{-1}\left(w\right)\right)-(1-\beta )D(1-w{)}^{\beta -1}\right],$$

and utilizing the complete sequence $\left\{(1-w{)}^{\left(n-2i\right)},n\geq 2i\right\}$, we can deduce that

$$f\left(F^{-1}(w)\right)=[\left(1-\beta \right)D{]}^{\frac{1}{\beta -1}}\left(1-w\right),\;a.e.w\in \left(0,1\right).$$

This implies that

(29) $$\frac{dF^{-1}(w)}{dw}=\frac{1}{f\left(F^{-1}(w)\right)}=\frac{1}{\left[(1-\beta )D{]}^{\frac{1}{\beta -1}}(1-w\right)}.$$

By solving this equation, it yields $F^{-1}(w)=\frac{-\log\left(1-w\right)}{[(1-\beta )D{]}^{\frac{1}{\beta -1}}}+d$, where d is an arbitrary constant. Utilizing the boundary condition $\underset{w\to 0}{lim}\;F^{-1}(w)=0$, it follows that d=0, we determine that d=0. Consequently, leading to $F^{-1}\left(w\right)=[-\log\left(1-w\right)]/[(1-\beta )D{]}^{\frac{1}{\beta -1}}$ for w>0. This implies the cdf $F(w)=1-e^{-{\left[\beta (1-\beta )H_{\beta }(T)+\beta \right]}^{\frac{1}{\beta -1}}w},w>0$, confirming that T follows an exponential distribution with scale parameter ${\left[\beta (1-\beta )H_{\beta }(T)+\beta \right]}^{\frac{1}{\beta -1}}$. This establishes the theorem. □

## 4. Tsallis Entropy-Based Exponentiality Testing

In this section, we propose a nonparametric method for estimating the Tsallis entropy of consecutive *k*-out-of-*n* good systems. Given the wide applicability of the exponential distribution in reliability and lifetime modeling, numerous test statistics have been developed to assess exponentiality—many of which are grounded in core principles of statistical theory. The primary objective here is to test whether the distribution of a random variable *T* follows an exponential law. Let $F_{0}(t)=1-e^{-\lambda t}$, for t>0, denote the cumulative distribution function under the null hypothesis. The hypothesis to be tested is formally stated as follows:

$$H_{0}:F\left(t\right)=F_{0}\left(t\right),vs.H_{1}:F\left(t\right)\neq F_{0}\left(t\right).$$

A specific case of Tsallis entropy of order 2, referred to as extropy, has recently attracted considerable attention as a useful metric for goodness-of-fit testing. Qiu and Jia [37] pioneered the development of two consistent estimators for extropy based on the concept of spacings and subsequently introduced a goodness-of-fit test for the uniform distribution using the more efficient of the two estimators. In a related contribution, Xiong et al. [38] utilized properties of classical record values to derive a characterization result for the exponential distribution, leading to the development of a novel exponentiality test. Their study outlined the test statistic in detail and demonstrated its effectiveness, particularly in small-sample scenarios. Building on this foundation, Jose and Sathar [39] proposed a new test for exponentiality based on a characterization involving the extropy of lower *r*-record values. Extending these developments, the present section explores the Tsallis entropy of consecutive *k*-out-of-*n* good systems. As established in Theorem 8, the exponential distribution can be uniquely characterized through the Tsallis entropy associated with such systems. Leveraging Equation (25), and following appropriate simplification, we now propose a new test statistic for exponentiality, denoted by $TS_{i,n,\beta }$, defined for n≥2i as follows:

(30) $$TS_{i,n,\beta }=H_{\beta }\left(T_{n-i|n:G}\right)-\eta_{i,n,\beta }\left[\beta H_{\beta }\left(T\right)+\frac{\beta }{1-\beta }\right]+\frac{1}{1-\beta },$$

where

$$\eta_{i,n,\beta }=\frac{[(n-i)(i+1){]}^{\beta (n-i+1)}}{[i(n-i+1){]}^{\beta (n-i)}}B\left(\frac{i(n-i+1)}{\left(n-i)(i+1\right)};\beta (n-i),\;\beta +1\right).for\;all\;\beta \in \Theta .If\;n\geq 2i,$$

then Theorem 8 directly implies that $TS_{i,n,\beta }=0$ if and only if T is exponentially distributed. This fundamental property establishes $TS_{i,n,\beta }$ as a viable measure of exponentiality and a suitable candidate for a test statistic. Given a random sample $T_{1},T_{2},\ldots ,T_{N}$, an estimator ${\hat{TS}}_{i,n,\beta }$ of $TS_{i,n,\beta }$ can be used as a test statistic. Significant deviations of ${\hat{TS}}_{i,n,\beta }$ from its expected value under the null hypothesis (i.e., the assumption of an exponential distribution) would indicate non-exponentiality, prompting the rejection of the null hypothesis. Consider a random sample of size N, denoted by $T_{1},T_{2},\ldots ,T_{N}$ drawn from an absolutely continuous distribution F and $T_{1:N}\leq T_{2:N}\leq \cdots \leq T_{N:N}$ represent the corresponding order statistics. To estimate the test statistic, we adopt an estimator proposed by Vasicek [40] for $\frac{dH^{-1}(w)}{dw}=1/h\left(H^{-1}(w)\right)$ as follows:

$$\frac{dF^{-1}(w)}{dw}≃\frac{N\left(T_{\left(l+m\right)}-T_{\left(l-m\right)}\right)}{2m},\;for\;all\;l=m+1,m+2,\ldots ,N-m,\;$$

and m is a positive integer smaller than N/2 which is known as the window size and $X_{l-m:N}=X_{1:N}$ for l≤m and $X_{l+m:N}=X_{N:N}$ for l≥N−m. So, a reasonable estimator for ${\hat{TS}}_{i,n,\beta }$ can be derived using Equation (30) as follows:

(31) $${\hat{TS}}_{i,n,\beta }=\frac{1}{N(1-\beta )}\sum_{l=1}^{N}\;{\left(\frac{2m}{N\left(T_{\left(l+m\right)}-T_{\left(l-m\right)}\right)}\right)}^{\beta -1}\left[{\left(k_{n-i|n:G}\left(\frac{l}{N+1}\right)\right)}^{\beta }-\beta \eta_{i,n,\beta }\right]\;.$$

Establishing estimator consistency is a fundamental step when evaluating estimators for parametric functions. The following theorem confirms the consistency of the estimator defined in Equation (31). The proof follows an approach similar to that of Theorem 1 in Vasicek [40], who introduced a widely adopted technique for proving consistency in entropy-based statistics. This method has also been employed by Park [41] and Xiong et al. [38] to validate the reliability of their respective test statistics.

**Theorem 9.** 
*Assume that $T_{1},T_{2},\ldots ,T_{N}$ is a random sample of size N taken from a population with pdf f and cdf F. Also, let the variance of the random variable be finite. Then ${\hat{TS}}_{i,n,\beta }\to^{p\;}$ $TS_{i,n,\beta }$ as N→+∞,m→+∞ and $\frac{m}{N}\to 0$, where $\to^{p\;}$ stands for the convergence in probability for all β∈Θ*.

**Proof.**  To establish the consistency of the estimator ${\hat{TS}}_{i,n,\beta }$, we employ the approach given in Noughabi and Arghami [42]. As both m and N tend to infinity, with the ratio m/N approaching 0, we can approximate the density as follows:

$$\begin{array}{c} \frac{2m}{N\left(X_{l+m:N}-X_{l-m:N}\right)}=\frac{F_{N}\left(X_{l+m:N}\right)-F_{N}\left(X_{l-m:N}\right)}{X_{l+m:N}-X_{l-m:N}}≃\frac{F\left(X_{l+m:N}\right)-F\left(X_{l-m:N}\right)}{X_{l+m:N}-X_{l-m:N}}\\ ≃\frac{f\left(X_{l+m:N}\right)-f\left(X_{l-m:N}\right)}{2}≃f\left(X_{l:N}\right),\;\;\;\; \end{array}$$

where $F_{N}$ represents the empirical distribution function. Furthermore, given that $\frac{l}{N+1}=$ $F_{N}\left(X_{l:N}\right)$, we can express

$$\begin{array}{c} {\hat{TS}}_{i,n,\beta }\;=\frac{1}{N(1-\beta )}\sum_{l=1}^{N}{\left(\frac{2m}{N\left(T_{\left(l+m\right)}-T_{\left(l-m\right)}\right)}\right)}^{\beta -1}\left[{\left(\rho_{n-i|n}\left(\frac{l}{N+1}\right)\right)}^{\beta }-\beta \eta_{i,n,\beta }\right]\\ \;\;\;\;\;\;\;\;≃\frac{1}{N(1-\beta )}\sum_{l=1}^{N}f^{\beta -1}\left(X_{l:N}\right)\left[{\left(\rho_{n-i|n}\left(F_{N}\left(X_{l:N}\right)\right)\right)}^{\beta }-\beta \eta_{i,n,\beta }\right]\\ \;\;\;\;\;\;\;\;\;=\frac{1}{N(1-\beta )}\sum_{l=1}^{N}f^{\beta -1}\left(X_{l:N}\right)\left[{\left(\rho_{n-i|n}\left(F\left(X_{l:N}\right)\right)\right)}^{\beta }-\beta \eta_{i,n,\beta }\right]\\ \;\;\;\;\;\;\;\;=\frac{1}{N(1-\beta )}\sum_{l=1}^{N}f^{\beta -1}\left(X_{l}\right)\left[{\left(\rho_{n-i|n}\left(F\left(X_{l}\right)\right)\right)}^{\beta }-\beta \eta_{i,n,\beta }\right]\;\;,\; \end{array}$$

where the second approximation relies on the almost sure convergence of the empirical distribution function i.e., $F_{N}\left(X_{l:N}\right)\to^{a.s.}F\left(X_{l:N}\right)$ as N→∞. Now, applying the Strong Law of Large Numbers, we have

$$\begin{array}{l} \frac{1}{N(1-\beta )}\sum_{l=1}^{N}f^{\beta -1}\left(X_{l}\right)[{\left(\rho_{n-i|n}\left(F\left(X_{l}\right)\right)\right)}^{\beta }\\ \;\;\;\;\;\;\;\;-\beta \eta_{i,n,\beta }]\;\;\;\to^{\;a.s.\;\;}\;E\left(\frac{1}{\left(1-\beta \right)}f^{\beta -1}(X)\left[{\left(\rho_{n-i|n}(F(X))\right)}^{\beta }-\beta \eta_{i,n,\beta }\right]\right)\\ \;\;\;\;\;\;\;\;=\frac{1}{\left(1-\beta \right)}\int_{0}^{\infty }f^{\beta }(w)\left[{\left(\rho_{n-i|n}(F(w))\right)}^{\beta }-\beta \eta_{i,n,\beta }\right]\;\;dw\\ \;\;\;\;\;\;\;\;=S_{\beta }\left(T_{n-i|n:G}\right)-\eta_{i,n,\beta }\left[\beta H_{\beta }(T)+\frac{\beta }{1-\beta }\right]+\frac{1}{1-\beta }, \end{array}$$

This convergence demonstrates that ${\hat{TS}}_{i,n,\beta }$ is a consistent estimator of $TS_{i,n,\beta }$ and hence completes the proof of consistency for all β∈Θ. □

The root mean square error (RMSE) of the estimator ${\hat{TS}}_{i,n,\beta }$ i is invariant under location shifts in the random variable *T*, but not under scale transformations. This property is formally established in the following theorem by adapting the arguments of Ebrahimi et al. [43].

**Theorem 10.** 
*Assume that $T_{1},T_{2},\ldots ,T_{N}$ is a random sample of size N taken from a population with pdf h and cdfH and $Y_{j}=aT_{j}+b,a>0,b\in R$. Denote the estimators for $TS_{i,n,\beta }$ on the basis of $T_{j}$ and $Y_{j}$ with ${\hat{TS}}_{i,n,\beta }^{T}$ and ${\hat{TS}}_{i,n,\beta }^{Y}$, respectively. Then, the following properties apply:*

(i)
$E\left({\hat{TS}}_{i,n,\beta }^{Y}\right)=E\left({\hat{TS}}_{i,n,\beta }^{T}\right)/a$ *,*
(ii)
$Var\left({\hat{TS}}_{i,n,\beta }^{Y}\right)=Var\left({\hat{TS}}_{i,n,\beta }^{T}\right)/a^{2}$ *,*
(iii) $RMSE\left({\hat{TS}}_{i,n,\beta }^{Y}\right)=RMSE\left({\hat{TS}}_{i,n,\beta }^{T}\right)/a$*, for all* β∈Θ.

**Proof.**  It is not hard to see from (25) that

$$\begin{array}{l} {\hat{TS}}_{i,n,\beta }^{Y}\;=\frac{1}{N(1-\beta )}\sum_{l=1}^{N}\;{\left(\frac{2m}{N\left(Y_{\left(l+m\right)}-Y_{\left(l-m\right)}\right)}\right)}^{\beta -1}\left[{\left(\rho_{n-i|n}\left(\frac{l}{N+1}\right)\right)}^{\beta }-\beta \eta_{i,n,\beta }\right]\\ \;\;\;\;\;\;=\frac{1}{N(1-\beta )}\sum_{l=1}^{N}\;\;{\left(\frac{2m}{Na\left(T_{\left(l+m\right)}-T_{\left(l-m\right)}\right)}\right)}^{\beta -1}[{\left(k_{n-i|n:G}\left(\frac{l}{N+1}\right)\right)}^{\beta }\\ \;\;\;\;\;\;-\beta \eta_{i,n,\beta }]\;\;={\hat{TS}}_{i,n,\beta }^{T}/a.\;\;\;\; \end{array}$$

Hence, we complete the proof by applying the properties of the mean, variance, and RMSE of ${\hat{TS}}_{i,n,\beta }^{Y}={\hat{TS}}_{i,n,\beta }^{T}/a$. □

A variety of test statistics can be constructed by selecting different combinations of *n* and *i*. For computational implementation, we consider the case *n* = 3 and *i* = 1, which simplifies the evaluation of the expression in Equation (30).

The test statistic ${\hat{TS}}_{1,3,2}$ converges to zero asymptotically as the sample size N approaches infinity under the null hypothesis $H_{0}$. Conversely, under an alternative distribution with an absolutely continuous cdf F, it converges to a positive value as N→+∞. Consequently, for a finite sample size N, we reject the null hypothesis at a significance level α if the observed value of ${\hat{TS}}_{1,3,2}$ exceeds the critical value ${\hat{TS}}_{1,3,2}(1-\alpha )$. However, the asymptotic distribution of ${\hat{TS}}_{1,3,2}$ is analytically intractable due to its complex dependence on N and the window parameter m.

**Figure 2:**
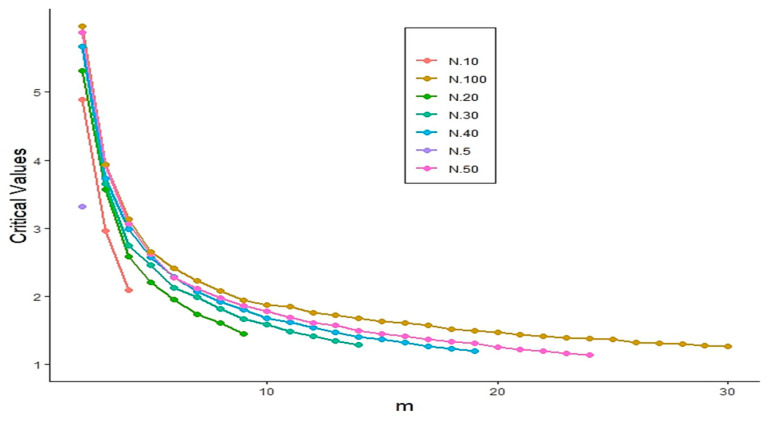
Critical values of the ${\hat{TS}}_{1,3,2}$ statistic at significance level α=0.05.

**Figure 3:**
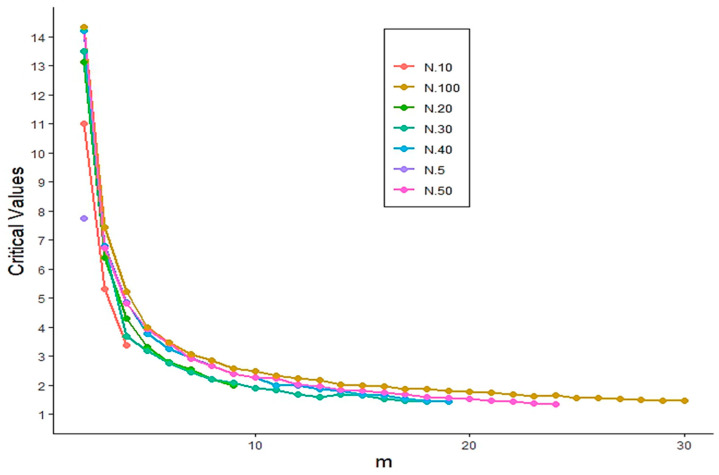
Critical values of the ${\hat{TS}}_{1,3,2}$ statistic at significance level α=0.01.

To address this challenge, we employed a Monte Carlo simulation approach. Specifically, 10,000 random samples of sizes N=5,10,20,30,40,50,100 were generated from the standard exponential distribution under the null hypothesis. For each sample size, we computed the $\left(1-\alpha \right)$-th quantile of the simulated values of ${\hat{TS}}_{1,3,2}$ to determine the critical values corresponding to significance levels α=0.05 and 0.01 while varying the window size *m* from 2 to 30. Figure 1 and Figure 2 display the resulting critical values for each sample size and significance level. The critical values of the proposed test statistic ${\hat{TS}}_{1,3,2}$ at the significance level α=0.01 are presented in Figure 3, which serve as the basis for evaluating the power performance through Monte Carlo simulations.

### Power Comparisons

The power of the test statistic ${\hat{TS}}_{1,3,2}$ was evaluated through a Monte Carlo simulation involving nine alternative probability distributions. For each specified sample size N=10,000 replicates of size N were drawn from each alternative distribution, and the value of ${\hat{TS}}_{1,3,2}$ was computed for each replicate. The empirical power at a given significance level α was estimated as the proportion of test statistics exceeding the corresponding critical value. To assess the efficiency of the newly proposed test based on ${\hat{TS}}_{1,3,2}$, its performance was benchmarked against several well-established tests for exponentiality reported in the literature. The specifications of the alternative distributions considered are summarized in Table 2.

The simulation setup, including the selection of alternative distributions and their associated parameters, closely follows the framework proposed by Jose and Sathar [39]. To evaluate the effectiveness of the newly proposed test based on the statistic ${\hat{TS}}_{1,3,2}$, its performance is compared with several well-established tests for exponentiality documented in the literature. A summary of these comparative tests is provided in Table 3.

The performance of the test statistic ${\hat{TS}}_{1,3,2}$ is influenced by the choice of window size *m*, making it necessary to determine an appropriate value in advance to ensure sufficient adjusted statistical power. Simulation results across various sample sizes led to the empirical recommendation m=⌊0.4N⌋, where ⌊x⌋ denotes the floor function. This heuristic formula offers a practical guideline for selecting *m* and aims to ensure robust power performance across a range of alternative distributions. To comprehensively evaluate the performance of the proposed ${\hat{TS}}_{1,3,2}$ test, we selected ten established tests for exponentiality and assessed their power against a diverse set of alternative distributions. Notably, Xiong et al. [38] proposed a test based on the Tsallis entropy of classical record values, while Jose and Sathar [39] introduced a test statistic using Tsallis entropy derived from lower *r*-records as a characterization of the exponential distribution.

The two tests referred to as $D_{9}$ and $D_{10}$ in Table 4 are included in our comparative analysis due to their basis in information-theoretic principles. The original authors provided extensive justification for their use in testing exponentiality, highlighting their theoretical soundness and practical applicability. To estimate the power of each test, we simulated 10,000 independent samples for each sample size N∈{10,20,50} from each alternative distribution specified in Table 4. The power of the proposed ${\hat{TS}}_{1,3,2}$ test was then computed at the 5% significance level. Subsequently, power values for both ${\hat{TS}}_{1,3,2}$ and the eleven competing tests were obtained using the same simulation framework. A summary of the comparative results is presented in Table 4.

Overall, the test statistic ${\hat{TS}}_{1,3,2}$ exhibits strong discriminatory power in detecting departures from exponentiality in the direction of the gamma distribution. In contrast, its performance against other alternatives, such as the Weibull, uniform, half-normal, and log-normal distributions, is more moderate, reflecting a balanced sensitivity without displaying either pronounced strength or notable limitations.

## 5. Conclusions

This study has investigated the utility of Tsallis entropy as a flexible and informative measure of uncertainty within the reliability framework of consecutive *k*-out-of-*n* good systems. A central contribution lies in establishing a meaningful relationship between the Tsallis entropy of such systems, under general continuous lifetime distributions, and their counterparts governed by the uniform distribution. Given the analytical complexity involved in deriving closed-form entropy expressions, especially for systems with large *n* or heterogeneous component behaviors, we derived a suite of informative bounds. These approximations not only facilitate practical computation but also deepen the theoretical understanding of entropy dynamics in complex system structures. Additionally, we proposed a nonparametric estimator specifically tailored to the structure of consecutive *k*-out-of-*n* systems. Its consistency and performance were validated through simulation studies and empirical applications. This estimator provides a valuable tool for quantifying system-level uncertainty and supports broader applications such as statistical inference and pattern recognition, including image processing and reliability-centered decision-making. In summary, this work contributes to the growing literature on information-theoretic measures in reliability by (i) establishing theoretical foundations that link Tsallis entropy to system reliability behavior; (ii) introducing practical bounding techniques to overcome analytical intractability; and (iii) developing a robust entropy-based estimator suitable for practical use.

Despite these advancements, several promising avenues remain open for further exploration: (i) The current analysis assumes independent and identically distributed components. Extending the framework to systems with dependent or heterogeneous lifetimes, such as those governed by copula-based models or frailty structures, would significantly broaden applicability. (ii) Investigating Tsallis entropy in more general system configurations (e.g., coherent systems, phased-mission systems, or dynamic networks) could yield new insights into uncertainty and resilience. (iii) Developing online or censored-data versions of the Tsallis entropy estimator would enhance its relevance in real-world reliability monitoring and predictive maintenance applications. (iv) Leveraging entropy measures to guide optimal system design (e.g., maximizing reliability for a fixed entropy budget) represents a novel and practically important direction. (v) A systematic comparison between Tsallis, Rényi, and cumulative residual entropy within the same system contexts may reveal cases where one measure is superior in inference, diagnostics, or optimization. These directions highlight the richness of Tsallis entropy as both a theoretical construct and a practical tool in reliability analysis and statistical modeling.

## Figures and Tables

**Table 1:** Notational Conventions and Their Definitions in the Context of Entropy Measures.

Symbol	Conceptual Definition	Location of Mathematical Definition	Alternative Notations in the Literature
S	Reliability function	N/A	$\bar{F},\bar{H}$
$S_{k|n}$	Reliability function of consecutive k-out-of-n good system	Equation (1), before Equation (2)	$S_{k|n:G},{\bar{F}}_{k|n:G},{\bar{H}}_{k|n:G}$
H	Shannon entropy	Equation (3), before Equation (4), after Equation (2)	S , h , H
$H_{\beta }$	Tsallis entropy	Equation (2), before Equation (3), after Equation (1)	$S_{q},S_{\beta },S_{\alpha },H_{\beta },H_{\alpha }$
$R_{\beta }$	Rényi entropy	Equation (6), after Equation (5)	$S_{\beta },S_{\alpha },H_{\beta },H_{\alpha }$

**Table 2:** Alternative Probability Distributions for Evaluating the Power of the Test Statistic.

Distribution	Probability Density Function	Support	Notation
Weibull	$f(t)=\frac{\alpha }{\beta }{\left(\frac{t}{\beta }\right)}^{\alpha -1}e^{-{\left(\frac{t}{\beta }\right)}^{\alpha }}$,	t>0,β,σ>0	W(α,β)
Gamma	$f(t)=\frac{1}{\beta^{\alpha }\Gamma (\alpha )}t^{\alpha -1}e^{-t/\beta }$	t>0,α,β>0	G(α,β)
Uniform	$f(t)=\frac{1}{\beta -\alpha }$,	α≤t≤β	U(α,β)
Half-Normal	$f(t)=\frac{\sqrt{\beta }}{\lambda \sqrt{\pi }}e^{-\frac{t^{\beta }}{\beta \lambda^{\beta }}}$,	t>0,λ>0	HN(λ)
Log-Normal	$f(t)=\frac{1}{t\lambda \sqrt{\beta \pi }}e^{-\frac{(lnt-\mu {)}^{2}}{\beta \lambda^{\beta }}}$,	t>0,λ>0,μ∈R	LN(μ,λ)

**Table 3:** Competing Tests for Exponentiality.

Test	Reference	Notation
1	Fortiana and Grané [44]	$D_{1}$
2	Choi et al. [45]	$D_{2}$
3	Mimoto and Zitikis [46]	$D_{3}$
4	Volkova [47]	$D_{4}$
5	Zamanzade and Arghami [48]	$D_{5}$
6	Baratpour and Rad [49]	$D_{6}$
7	Noughabi and Arghami [50]	$D_{7}$
8	Volkova and Nikitin [51]	$D_{8}$
9	Torabi et al. [52]	$D_{9}$
10	Xiong et al. [38]	$D_{10}$
11	Jose and Sathar [39]	$D_{11}$

**Table 4:** Power comparisons of the tests at the significance level α=0.05.

N	$H_{1}$	$D_{1}$	$D_{2}$	$D_{3}$	$D_{4}$	$D_{5}$	$D_{6}$	$D_{7}$	$D_{8}$	$D_{9}$	$D_{10}$	$D_{11}$	${\hat{TS}}_{1,3,2}$
10	G(1,1)	5	5	5	5	5	5	5	5	5	5	5	5
	G(0.4, 1)	34	11	50	46	29	7	0	50	65	60	83	75
	W(1.4, 1)	15	17	16	13	16	23	29	16	1	15	17	3
	HN(1)	11	10	10	8	10	8	20	10	1	18	8	5
	U(0, 1)	42	28	31	15	33	60	51	24	2	93	100	10
	LN(0, 0.8)	12	24	16	21	23	17	26	19	2	5	5	2
	LN(0, 1.4)	36	19	40	6	29	15	1	9	47	3	2	3
20	G(1, 1)	5	5	5	5	5	5	5	5	6	5	5	5
	G(0.4, 1)	56	31	77	80	66	25	0	82	89	95	99	96
	W(1.4, 1)	32	27	34	27	17	33	47	29	6	29	8	2
	HN(1)	23	14	19	12	7	30	23	14	2	37	5	4
	U(0, 1)	86	52	63	28	54	92	80	39	18	100	100	12
	LN(0, 0.8)	18	52	26	48	42	18	49	45	8	4	4	0
	LN(0, 1.4)	61	45	67	11	64	43	0	16	71	0	3	2
50	G(1, 1)	5	5	5	5	5	5	5	5	5	5	5	5
	G(0.4, 1)	89	78	99	99	95	68	0	99	100	100	100	100
	W(1.4, 1)	73	55	79	65	5	62	80	67	37	56	7	0
	HN(1)	59	23	52	25	1	54	48	28	13	73	2	2
	U(0, 1)	100	91	98	62	62	100	99	72	78	100	100	24
	LN(0, 0.8)	26	93	44	93	55	24	84	92	47	3	3	0
	LN(0, 1.4)	93	85	95	25	93	85	0	29	95	0	2	0

## Data Availability

The datasets are available within the manuscript.
